# Evaluation of long-term stability of mesiodistal axial inclinations of maxillary molars through panoramic radiographs in subjects treated with Pendulum appliance

**DOI:** 10.1590/2177-6709.21.1.067-074.oar

**Published:** 2016

**Authors:** Caroline Andrade Rocha, Renato Rodrigues de Almeida, José Fernando Castanha Henriques, Carlos Flores-Mir, Marcio Rodrigues de Almeida

**Affiliations:** 1 MSc, Universidade de São Paulo (USP), School of Dentistry, Department of Orthodontics, Bauru, São Paulo, Brazil; 2 Associate Professor, Universidade de São Paulo (USP), School of Dentistry, Department of Orthodontics, Bauru, São Paulo, Brazil. Full Professor, Universidade Norte do Paraná (UNOPAR), Department of Orthodontics, Londrina, Paraná, Brazil; 3 Full Professor, Universidade de São Paulo (USP), School of Dentistry, Department of Orthodontics, Bauru, São Paulo, Brazil; 4 Professor and Head Division of Orthodontics, University of Alberta, Edmonton, Alberta, Canada; 5 Full Professor, Universidade Norte do Paraná (UNOPAR), Department of Orthodontics, Londrina, Paraná, Brazil

**Keywords:** Orthodontic appliances, Angle Class II malocclusion, Radiography

## Abstract

**Objective:**

To evaluate the stability of mesiodistal inclination of maxillary molars produced by a pendulum appliance, five years after completion of orthodontic treatment. Angulation changes were compared to an untreated sample.

**Methods:**

The sample consisted of 20 patients (14 females and 6 males) with Class II, Division 1 malocclusion that was treated through molar distalization with a pendulum appliance followed by cervical headgear and full fixed appliances. Maxillary molar inclination was evaluated through panoramic radiograph. The mean age at pretreatment was 14.3 ± 1.6 years, whereas at immediate post-treatment it was 18.6 ± 1.8 years, and at long-term post-treatment it was 23.8 ± 2.0 years. A control group of 16 untreated individuals with untreated normocclusion ranging in age from 12 to 17 years old were used as comparison group. Data were statistically analyzed with independent t-tests and ANOVA test followed by Tukey post-hoc tests.

**Results:**

Statistically significant differences were found between T_1_(94.5^0)^ and T_2_ (98.8^0)^ as well as between T_2_ and T_3_ (94.7^0)^ for maxillary first molars. Maxillary second molars did not show any statistically significant positional changes during the evaluated time periods T_1_ (107.5^0)^, T_2_ (109.3^0)^ and T_3_ (106.9^0)^.

**Conclusion:**

Although maxillary first molars underwent distal crown inclination immediately after treatment, approximately five years thereafter their roots tended to upright close to the pretreatment positions.

## Abstract (pt)

**Objetivo:**

avaliar a estabilidade da inclinação mesiodistal dos molares superiores cinco anos após o tratamento realizado com o aparelho Pendulum seguido de aparelho ortodôntico fixo. As mudanças na angulação foram comparadas com uma amostra não tratada.

**Métodos:**

a amostra consistiu de 20 pacientes (14 do sexo feminino e 6 do masculino) com má oclusão de Classe II, divisão 1 tratados por meio da distalização dos molares com o aparelho Pendulum seguido de ancoragem extrabucal cervical e aparelho fixo. A inclinação dos molares superiores foi avaliada por meio de radiografias panorâmicas. A média da idade ao início do tratamento foi de 14,3 ± 1,6 anos; ao final do tratamento, de 18,6 ± 1,8 anos e, no longo tempo pós-tratamento, de 23,8 ± 2,0 anos. Para comparação, foi usado um grupo controle com 16 indivíduos não tratados e com oclusão normal, apresentando idades entre 12 e 17 anos. Os dados foram analisados estatisticamente pelo teste *t* independente e ANOVA, seguido do teste de Tukey.

**Resultados:**

os primeiros molares superiores apresentaram diferenças estatisticamente significativas entre T_1_ (94,5^o)^ e T_2_(98,8^o)^, bem como entre T_2_ e T_3_(94,7^o)^. Os segundos molares superiores não apresentaram alterações estatisticamente significativas no seu posicionamento durante os períodos avaliados, T_1_ (107,5^o)^, T_2_(109,3^o)^ e T_3_ (106,9^o)^.

**Conclusões:**

apesar de os primeiros molares superiores apresentarem inclinação distal da coroa imediatamente após o tratamento, aproximadamente cinco anos depois suas raízes tendem a ocupar posições próximas às do início do tratamento.

## INTRODUCTION

In Class II malocclusion cases without a significant anteroposterior skeletal component, an available treatment option is to distalize maxillary molars and premolars to allow canines and incisors to attain normal overjet and overbite.^1 ^In the past,molar distalization was accomplished mainly with the use of headgears.^2^The objective was to obtain a bodily distal movement of maxillary first molars by adjusting the headgear, so that the line of force application passed through molars center of resistance. A problem with the use of headgears is their compliance dependency.^3^ Currently, there are other intraoral distalization appliances that are not compliant dependent.^4^^,^ 5 The reported disadvantage of these appliances is the potential loss of posterior anchorage during the overjet reduction stage. 

The pendulum appliance was developed in 1992 as a noncompliance intraoral molar distalization appliance. Several authors have shown its effectiveness in the correction of Class II molar relationships.^6^^-^ 9 One identified limitation is that the more distal molar movement is produced, the more crown tipping is observed. The explanation for this side effect is the off-centered location of the point of force application.^10^The importance of obtaining correct mesiodistal axial inclination with parallel roots is often emphasized in the literature.^11^^,^ 12 Improved occlusal stability by neutralization of occlusal forces is suggested.^13^

Panoramic radiography is frequently used to determine root parallelism and mesiodistal axial inclination of teeth in orthodontic practices.^14^^,^ 15 Although cone-beam volumetric tomography can provide more precise information, this method is not yet routinely available.^16^ Moreover, panoramic radiograph is less expensive and more commonly performed, since it produces less radiation exposure.^17^ It can be argued that craniofacial structures are magniﬁed and distorted in panoramic radiographs;^18^however, some studies point out that relative accuracy can be obtained with the use of panoramic radiographs to assess angular measurements, if proper precautions are taken when positioning the head.^19^^,^ 20

There is scarcity of long-term studies evaluating the stability of dental changes produced by the pendulum appliance. Only two studies have evaluated the long-term stability of treatment effects with the pendulum appliance.^21^^,^ 22Significant dentoalveolar effects, as measured through lateral cephalograms, were partially maintained after a follow-up period of seven years.^21^ Partial stability was also shown when measuring dentoalveolar variables through the PAR index five years after treatment.^22^ Therefore, the objective of this study was to evaluate, through panoramic radiographs, the long-term stability of mesiodistal axial inclinations of maxillary molars after orthodontic treatment with the pendulum appliance followed by a cervical headgear and fixed orthodontic appliances, as compared to the expected normal values determined from an untreated sample. To this end, the null hypothesis assumed that mesiodistal axial maxillary molar inclination was not affected in the long term by treatment with the pendulum appliance followed by a cervical headgear and fixed orthodontic appliances.

## MATERIAL AND METHODS

Material

**Figure 1:**
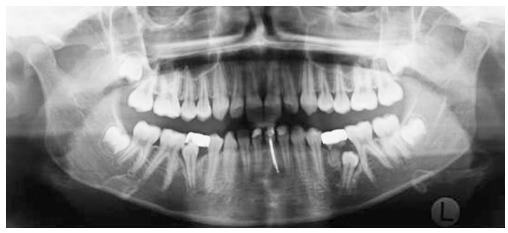
- Pretreatment (T_1_) panoramic radiograph.

**Figure 2:**
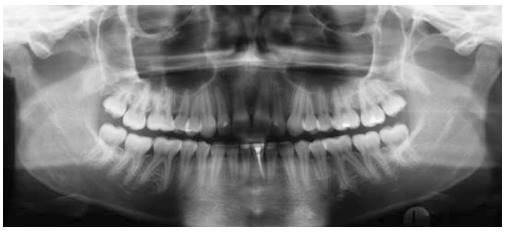
- Post-treatment (T_2_) panoramic radiograph.

**Figure 3:**
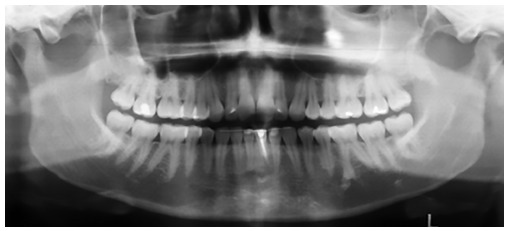
- Long-term post-treatment (T_3_) panoramic radiograph.

Ethical approval was granted by Universidade de São Paulo (USP) Bauru School of Dentistry. In this retrospective clinical study, no sample size calculation was done as all available records were considered. From the available 33 patients treated with the pendulum appliance followed by a cervical headgear and fixed appliances, 11 patients' records were later excluded for the following reasons: three patients had their pendulum appliances removed prematurely due to breakage; three patients had inadequate radiographs because of improper head positioning at the time of exposure; and five had incomplete records. Out of the remaining 22 patients, two did not return for long-term post-treatment records (five years or more). Therefore, the final available sample consisted of 20 patients (6 males, 14 females). The initial mean pretreatment age was 14.3 ± 1.6 years. Thus a total of 60 panoramic radiographs was available from pretreatment (T_1_), immediate post-treatment (T_2_) and long-term post-treatment (T_3_) records (Figs 1-3).

All patients met the following inclusion criteria: (1) Angle Class II molar relationship (12 patients had full Class II molar relationships and eight had one-half Class II molar relationships in either side); (2) presence of all permanent teeth, including fully erupted maxillary second molars.

These patients were assessed on an average of 5.2 years after the end of orthodontic treatment (T_3_). At T_1_, 19 patients had unerupted third molars present while the remaining patients had agenesis of third molars. At T_2_, only one patient had extraction of third molars. Finally, at T_3_, only four additional patients had third molars extracted.

A control group of 16 untreated normocclusion individuals ranging in age from 12 to 17 years were used as comparison group. Their maxillary molar mesiodistal angulations were considered normal for the purposes of this study. They had a full complement of teeth (except for third molars), Class I canine and molar relationship, overbite and overjet between 1 and 3 mm and absence of crowding. This control group, used previously by other researchers,^14^^,^ 15 was obtained from the files of the Universidade de São Paulo (USP) Bauru School of Dentistry Growth Study.

### Appliance setup

Each patient received a pendulum appliance, as described in the literature.^4 ^All orthodontic treatment was managed by one clinician and consisted of an acrylic Nance button and two coil springs (0.032-in TMA wire, Ormco, Glendora, CA, USA). Each pendulum was anchored to the first premolar through bands and to the second premolar through wires bonded to the occlusal surfaces. The pendulum springs were activated parallel to the palatal midline, with a mean force of about 250 g. Mean treatment time was 5.9 ± 1.8 months, until an overcorrected Class I molar relationship was achieved. The mean space opened was 7.25 mm, as determined through lateral cephalograms, and on the dental casts, the mean space opening on the right and left arch sides were 6.12 and 6.5 mm, respectively. Thereafter, the pendulum appliance was removed and a Nance button was used to anchor and retain the distalized maxillary first molars. Anchorage reinforcement was achieved with a cervical headgear with the outer bows tilted 15° to 20° upward from the occlusal plane, exerting 400 to 500 g of force with an average wear of 10 to 12 hours per day. 

Following distalization of first molars, preadjusted Edgewise orthodontic appliances were bonded to finish occlusal detailing. The archwire sequence was: nickel-titanium 0.016-in, stainless steel 0.018-in, 0.020-in, and 0.019 x 0.025-in. During the use of the 0.019 x 0.025-in rectangular arch, sequential retraction of second premolars, followed by first premolars, was initiated with intraoral elastics while the cervical headgear was worn at night. After retraction of first premolars, the Nance button was removed for mass retraction of anterior teeth. At this stage, in addition to the cervical headgear, Class II elastics (12 to 20 hours per day) were used for retention of the molar relationship and reduction of overjet. After retraction of maxillary anterior teeth, finishing archwires were placed for completion of treatment. After removal of the fixed orthodontic appliances, standard upper Hawley and lower 3 x 3 fixed retainers were placed. Total treatment time (considering the pendulum, cervical headgear and fixed appliances use) was 4.4 ± 0.8 years, while the long-term post-treatment evaluation was 5.2 ± 1.1 years. The breakage rate of pendulum appliance was 9.09%.

## Methods

Panoramic radiographs of experimental and control groups were taken using the same equipment Funk X-15 (Funk, Ribeirão Preto, SP, Brazil) with a cephalostat. Clinical Frankfort horizontal plane was parallel to the ground and the facial midline plane was perpendicular to the ground. 

For each patient in the experimental group, panoramic radiographs were taken at T_1_, T_2_ and T_3_. The mean period between T_1_ and T_2_ was 4.4 years and between T_2_ and T_3_ was 5.2 years. No additional panoramic radiographs were taken during treatment. For subjects in the control group, only one radiograph was used (12 to 17 years of age).

**Figure 4:**
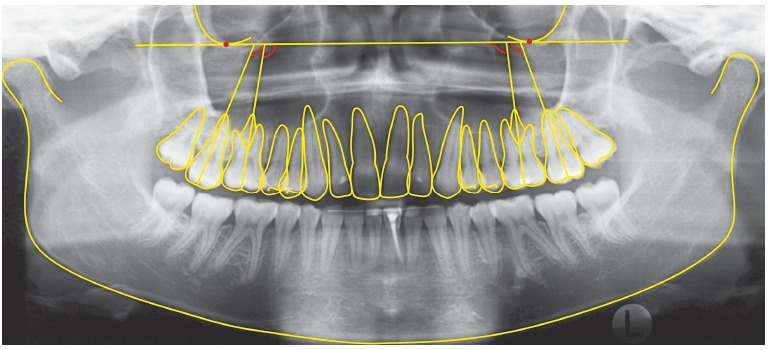
- Angular measurements between long axes of teeth and upper reference line.

The inferior outline of the orbits and the contours of maxillary molars were traced on acetate paper over each radiograph by one evaluator, while the tracings were verified by another evaluator. The reference line used passed through the most inferior points of the right and left orbits.^23^ The angles formed by the upper reference line and the long axes of teeth (palatal root) were measured on each radiograph. Figure 4 shows the anatomic structures, references lines and angular measurements used in this study.

### Error of the method

To assess the error of localizing the reference points and the manual procedure, 18 randomly selected radiographs were retraced and remeasured by the same examiner about four weeks later. Random errors were assessed by Dahlberg's formula, and the systematic errors were ascertained by paired t-tests. Intraobserver reproducibility of angular measurement was examined by Intraclass Correlation Coefficient (ICC). The degree of concordance observed is classified as: weak if ICC is < 0.4, satisfactory ≤ 0.4 and < 0.75 and excellent ≥ 0.75.^24^

### Statistical analysis

Means and standard deviations for each variable were calculated. Normal distributions were verified by the Kolmogorov-Smirnov test. Results of this test showed that all variables were normally distributed. Therefore, paired ANOVA, followed by Tukey tests were used. The mean mesiodistal axial inclinations at stages T_1_, T_2_ and T_3_ were compared with normal mean values through independent t-test. All statistical analyses were performed with Statistica software (Statistica for Windows 7.0; Statsoft, Tulsa, Oklahoma, USA). Results were considered statistically significant at *p*< 0.05.

## RESULTS

Systematic and random errors varied from 0.91° (maxillary second left molar) to 1.50° (maxillary first right molar). Therefore, the random error of the method (Dahlberg's formula) did not exceed 1.50°. Paired t-tests did not show statistically significant differences for systematic errors (*p* < 0.05). Reproducibility was found to be excellent through ICC values of 0.838, 0.846, 0.932 and 0.968, respectively for maxillary first right molar, maxillary first left molar, maxillary second right molar, maxillary second left molar. 

The mean mesiodistal axial inclination values at T_1_, T_2_ and T_3_ and the significance of their differences are found on Table 1. Statistically significant differences were demonstrated between T_1_ and T_2_, as well as between T_2_ and T_3_ for maxillary first right and left molars.

**Table 1:** - Means and standard deviation values of tooth at pretreatment (T_1_), post-treatment (T_2_) and long-term post-treatment (T_3_), and results of dependent ANOVA and Tukey tests.

**Tooth**	**T_1_**	**T_2_**	**T_3_**	***p***			
	**Mean**	**SD**	**Mean**	**SD**	**Mean**	**SD**	
First maxillary right molar	94.45^a^	4.91	98.95^b^	5.50	95.25^a^	4.40	0.001866*
First maxillary left molar	94.5^5a^	7.54	98.60^b^	4.19	94.25^a^	5.38	0.011745*
Second maxillary right molar	108.10	7.66	109.40	7.24	107.75	8.47	0.584236
Second maxillary left molar	106.80	8.56	109.25	7.35	106.10	9.49	0.109888

At T_1_, no statistically significant differences between groups were demonstrated (Table 2). 

**Table 2:** - Independent t-test for groups at pretreatment (T_1_).

**Tooth**	**Control group**	**Experimental group**	***t***	***p***		
	**Mean**	**SD**	**Mean**	**SD**		
First maxillary right molar	91.75	3.47	94.45	4.91	-1.85547	0.072216
First maxillary left molar	92.75	3.64	94.55	7.54	-0.87534	0.387529
Second maxillary right molar	108.56	5.03	108.10	7.66	0.20803	0.836442
Second maxillary left molar	109.25	3.53	106.80	8.56	1.07213	0.291212

At T_2_, statistically significant differences between groups were demonstrated only for maxillary first right and left molars (Table 3).

**Table 3:** - Independent t-test for groups at post-treatment (T2).

**Tooth**	**Control group**	**Experimental group**	***t***	***p***		
	**Mean**	**SD**	**Mean**	**SD**		
First maxillary right molar	91.75	3.47	98.95	5.50	-4.55249	0.000065*
First maxillary left molar	92.75	3.64	98.60	4.19	-4.41006	0.000099*
Second maxillary right molar	108.56	5.03	109.40	7.24	-0.39239	0.697216
Second maxillary left molar	109.25	3.53	109.25	7.35	0.00000	1.000000

* Statistically significant at p < 0.05.

At T_3_, statistically significant differences between groups were demonstrated only for maxillary first right molar (Table 4).

**Table 4:** - Independent t-test for groups at long-term post-treatment (T_3_).

**Tooth**	**Control group**	**Experimental group**	***t***	***p***		
	**Mean**	**SD**	**Mean**	**SD**		
First maxillary right molar	91.75	3.47	95.25	4.40	-2.59743	0.013781*
First maxillary left molar	92.75	3.64	94.25	5.38	-0.95298	0.347324
Second maxillary right molar	108.56	5.03	107.75	8.47	0.338261	0.737246
Second maxillary left molar	109.25	3.53	106.10	9.49	1.256707	0.217425

* Statistically significant at *p* < 0.05.

## DISCUSSION

The aim of this study was to evaluate the mesiodistal axial inclination of maxillary molars, five years after completion of orthodontic treatment with a pendulum appliance followed by a cervical headgear and fixed appliances. It must be emphasized that the effectiveness of the pendulum appliance in the correction of Class II molar relationships has been previously reported.^6^^-^ 9 However, only two studies evaluated the stability of pendulum appliance correction.^21^^,^ 22 One of this studies evaluated the long-term stability (seven years) of molar movements following pendulum and fixed appliance and found no maxillary molar relapse during the postretention period.^21^ The other study^22^ evaluated treatment stability (five years) of the pendulum appliance followed by fixed appliances by means of PAR index in dental casts and cephalometric measurements. It was concluded that treatment was stable (PAR relapse percentage of only 17.19%). The results also showed great stability of cephalometric variables. 

Both studies evaluated, through a clinical cast analysis, the stability of molar position after pendulum appliance correction. Neither one used panoramic radiographs. Panoramic radiography is frequently used to assess root parallelism and mesiodistal axial inclination before, during, and after treatment.^12^^,^ 14 ^,^ 15 Although it can be argued that craniofacial structures are magniﬁed and distorted in panoramic radiographs,^12^^,^ 18 ^,^ 25 accurately assessing mesiodistal tooth inclination with panoramic radiograph is still possible, as suggested by other studies.^12^^,^ 20Although some authors^20^ emphasized that linear measurements were unreliable; angular measurements, such as axial tooth inclinations, are not as variable.^20^^,^ 26 To diminish distortion and magnification of images as much as possible, radiographs were carefully obtained through standard exposure parameters and proper patient posture. Small deviations of ideal head position can significantly affect panoramic radiograph; however, there is some tolerance of variation in head positioning.^19^^,^ 20 Studies demonstrate that variation of occlusal plane in 10 degrees or less do not significant affect teeth angulation.^19^^,^ 20

Regarding specific distortions in the molar area, in one study, a change of 5 degrees in inferior head tilt resulted in significant changes in maxillary molars inclination.^27^ In another study, maxillary posterior roots appear to be projected more distally in panoramic radiography.^18^ Nevertheless, panoramic radiograph should be used with caution to assess the axial inclination of teeth, considering its distortion and alteration due to aberrant head positioning or inherent distortion problems of the radiographic method.^18^^,^ 25

Evaluating the axial inclinations of teeth has potential significant relevance in Orthodontics. Orthodontic treatment objectives include obtaining functional occlusion, esthetics and stability. A criterion for obtaining functional occlusion is to have ideal axial inclinations of all teeth after active treatment. This is especially important for orthodontically closed extraction sites which are more likely to open if adjacent teeth roots are not parallel.^13^^,^ 23

In this sample, maxillary second molars were already erupted into the occlusal plane. It has been previously proposed that tipping of maxillary first molars was much more pronounced than bodily movement when maxillary second molars were still at the budding stage. In contrast, when eruption of maxillary second molar was completed, distalization of maxillary first molars happened almost exclusively by bodily movement.^9^ To further support this concept, a recent systematic review showed that the effect of maxillary second molar eruption stage on molar angular distalization appears to be minimal; however, the large variability reported should be considered clinically.^28^

Our results did not demonstrate statistically significant differences between the mean values at T_1_ from those with normal untreated occlusions. In this Class II malocclusion sample, molar inclination was acceptable at T_1_. Results later showed that treatment changed the mesiodistal axial inclinations of maxillary first molars through distalization of the molar crown (9.4^0^ of distal inclination immediately after distalization) without a similar degree of root distal movement. This crown distal inclination was expected, since the pendulum appliance, as other similar intraoral distalizers, tend to tip the molar distally due to the point of force application as related to the center of resistance.^7^^,^ 10 ^,^ 29 However, in this sample, molar inclination almost normalized at T_3_, indicating that either normal growth changes or dental relapse might have improved maxillary molar inclinations. It has been proposed that fully erupted permanent teeth can still move as part of normal craniofacial changes well into adulthood.^30^ This change is evidenced radiographically by previously reported mesial axial inclination of maxillary first and second molars.^30^ Although a possible hypothesis is that the horizontal component of force during chewing may cause a mesial axial inclination of molars while adapting to the occlusal functional demands,^31 ^the reported differences may not be clinically meaningful. Only the maxillary first right molar presented statistically significant differences at this stage. It has to be considered that this statistical difference was small and therefore considered clinically insignificant.

## Limitations

The relatively small sample size of both treatment and control groups should be considered. Additionally, the use of cross sectional data, instead of longitudinal data for the control group could be considered a limiting factor. Furthermore, the current method does not indisputably allow determination if the change in molar angulation is as a result of dental relapse or normal growth changes.

## CONCLUSIONS

In this sample, treatment with the pendulum appliance followed by a cervical headgear and fixed orthodontic appliances caused distal tipping of maxillary first molars (between 94.5^0^ and 98.8^0^), but in the long term, these axial inclination values tended to normalize to pretreatment values (between 98.8^0^ and 94.7^0^). Thus, the null hypothesis that mesiodistal axial maxillary molar inclination would not be affected by treatment with pendulum appliance followed by fixed orthodontic appliance in the long term was rejected.
